# Beyond medications: a multifaceted approach to alleviating comorbid anxiety and depression in clinical settings

**DOI:** 10.3389/fpsyg.2024.1456282

**Published:** 2024-09-02

**Authors:** Sofia Svensén, Ingeborg Bolstad, Liv Skomakerstuen Ødbehr, Gerry Larsson

**Affiliations:** ^1^Inland Norway University of Applied Sciences, Elverum, Norway; ^2^Swedish Defence University, Karlstad, Sweden

**Keywords:** depression, anxiety, comorbidity, treatment, physical activity, healthy diet, patient education, social support

## Abstract

**Background:**

Comorbid anxiety and depression are common and can make the problems more complex and sometimes resistant to pharmacological treatment. In existing research, the diagnoses are often studied separately, and physical activity, healthy nutrition, psychoeducation, and social support have shown good effects. The aim of the present study was to explore the longitudinal effects of a comprehensive treatment on patients with comorbid anxiety and depression in a clinical context.

**Method:**

Eighty inpatients (15 men and 65 women) in age range 23–65 years receiving psychiatric treatment in Norwegian clinic participated in the longitudinal study. Treatment was person-centered and was most frequently given for anxiety and depression, e.g., pharmacological treatment and psychotherapy, individually and in groups. In combination with this, physical activity, healthy nutrition, psychoeducation and social support in contacts with authorities and relatives were also a part of treatment. Depression and anxiety were assessed using the Beck Anxiety Inventory and Beck Depression Inventory at three points in time: baseline, at the end of treatment, and 3 months after treatment. The answers were categorized and combined into four groups according to severity of anxiety and depression to measure effects on comorbidity. Mann Whitney U test, Chi-square, Friedmans test, and McNemar test were used to analyze the data.

**Result:**

The results showed a significant increase of frequencies in the group with mild anxiety and depression 3 months past treatment compared to baseline.

**Conclusion:**

Through the comprehensive, person-centered treatment more patients had low levels of both anxiety and depression 3 months after treatment. We suggest that clinics working with comorbid depression and anxiety patients should add physical activity, nutrition advice, social support, and psychoeducation to the traditional treatment regimes. More research concerning comorbid anxiety and depression are urgent to further expand the treatment possibilities.

## 1 Introduction

Mental illness is a global health problem in increase, with anxiety and depression being the most common. The World Health Organization (WHO) estimates that approximately four per cent of the world's population suffers from anxiety and five per cent of the adult population suffers from depression, and there is a higher incidence of the diagnoses among women than men. The causes may be biological, psychological, or social and vary between individuals, and experiences of traumatic events are associated with an increased risk (WHO, 2023a,b). Anxiety and depression share many components and in clinical contexts, co-occurrence of depression and anxiety, so called comorbidity, is common, about 70 per cent of patients with depression or anxiety also show symptoms of the other diagnosis (Lamers et al., 2011; Dunner, 2001). However, in the extant research the diagnoses are mostly studied separately, not combined. Complementary treatments such as physical activity, healthy nutrition, psychoeducation and social support are also generally studied one and one and not combined. This study focuses on anxiety and depression comorbidity and a combined treatment program.

The Diagnostic and Statistical Manual of Mental Disorders 5th edition (DSM-5) states that to set the diagnosis of depression, the presence and severity of specific symptoms, such as low mood, cognitive problems, and inability to feel joy, should be present (American Psychiatric Association, 2013).

Anxiety means worry and fear of something that is perceived as threatening. The diagnosis is characterized according to DSM-5 by six symptoms: restlessness, muscle tension, sleep disturbances, lack of energy, irritation, and concentration difficulties. For a diagnosis, a presence of at least three of these symptoms is required (American Psychiatric Association, 2013).

The treatment of depression and anxiety often consists of pharmacological treatment and/or psychotherapy, such as cognitive behavioral therapy (CBT) with focus on reducing the symptoms (Goodwin, 2021; WHO, 2023a,b). Depression/anxiety comorbidity can make the problems more complex (Ironside et al., 2023; Zhiguo and Yiru, 2014; Chen, 2022) and patients less receptive to traditional treatment, in some cases even resistant to pharmacological treatment (Zhiguo and Yiru, 2014). In recent years, however, interest in treatment of depression and anxiety that takes several dimensions of the individual into account has increased, as well as insights into how mental health is affected by, among other things, physical activity, good nutrition, and social relationships. Such lifestyle changes may not only lead to better physical health, but it also give a sense of control and self-efficacy to be able to influence one's own mood and situation (Rolin et al., 2020). Through such changes, the individual can experience increased comprehensibility, manageability, and meaningfulness (Antonovsky, 1987), which in the long-term may induce a feeling of control and a stress reduction.

Physical activity has been shown to have good effects on mainly depression but also anxiety (Andersson et al., 2022; Merom et al., 2008; Singh et al., 2023). In mild or moderate depression, effects are as good as for pharmacological treatment (Andersson et al., 2015; Cooney et al., 2013). Physical activity is an easily accessible, cost-effective treatment option bringing small risks of side effects, but can have good effects on mental and physical health (Hearing et al., 2016).

When it comes to the effect of physical activity on mental health in comorbid depression and anxiety, the relationship is more unclear. A systematic review by Bond et al. (2020) showed that no studies concerning the effect of physical activity focused on comorbidity. Where comorbidity was present, results were focused on the effects on depression symptoms. Researchers urge the importance of further exploring the effects of physical activity and nutrition on comorbid anxiety/depression. As the diagnoses often occur together it is of the utmost importance to see the diagnoses in context and not to distinguish them in treatment (Gibson-Smith et al., 2018; Rebar et al., 2017). The fact that comorbidity is often not included in studies, focus is on either depression or anxiety, leads to a lower ecological validity (Rebar et al., 2017).

The importance of social support for maintaining a good mental health is well known (Beauregard et al., 2011; Shao et al., 2020; Terry and Townley, 2019) and may decrease the prevalence of depression or anxiety (Grey et al., 2020). A study including six randomized controlled trials (RCT) showed that perception of higher degrees of social support increased the effects of treatment of major depression (Buckman et al., 2021). Loneliness and an absence of social support may also affect eating habits (Zhang et al., 2024; Hanna et al., 2023).

There are also associations between the two diagnoses and poor nutrition habits (Gibson-Smith et al., 2018; Zhiguo and Yiru, 2014). One reason for poor nutrition in mental illness can be, so called, emotional eating, that is, a way of dealing with stress and difficult feelings. Eating may be a way to self-medicate like what is seen for other types of addiction (Roer et al., 2021). Poorer dietary habits and obesity can also be caused by various pharmacological treatments as a side effect (Theal et al., 2018).

A 3-month long intervention study in which individuals with moderate to severe depression participated showed significant larger positive effects in the nutrition group compared to the control group (Owen and Corfe, 2017) and systematic reviews have concluded that a healthy diet could decrease the risks for depression (Lai et al., 2014; Li et al., 2017).

Anxiety is also related to unhealthy nutrition and research have shown associations to sweet craving as well as emotional eating (de Oliveira Penaforte et al., 2019). How anxiety and depression affect a person's relation to food and appetite may vary. Paans et al. (2018) showed an association between depression and emotional eating where more severe depression led to an increase in emotional eating. On the other hand, Simmons et al. (2016) concluded that depression may cause a loss of appetite, and the same effect has been shown for anxiety (Leonard, 2020). Though, to summarize, anxiety and depression often led to a problematic relationship to nutrition and food.

The use of patient education, often called psychoeducation, might be a way to empower the patient to understand and cope with the symptoms. A systematic review conducted by Lean et al. (2019) investigated the effects of self-management interventions that included psychoeducation for individuals with severe mental illness. The authors concluded that, in combination with traditional treatment, the intervention could increase the effects for individuals with severe mental illness.

A study that included all above-mentioned inventions, and some more, was conducted by Rolin et al. (2020). Participants with self-reported mental illness (a majority rated depression or anxiety as the primary psychiatric diagnosis) completed a 90-day self-management mental health program that included physical activity, meditation and a focus on sleep, healthy nutrition, and social contact. During the first 30 days participants were requested to follow healthy lifestyle recommendations in a workbook, and then keep going by themselves for the remaining 60 days. At follow-up after the first 30 days, the study showed improvements in symptoms of depression and anxiety, sleep quality, perceived optimism, and resilience. No further follow-up analysis after 90 days was performed. This is in line with a study conducted by Van Citters et al. (2010) were participants with different types of severe mental illness, with a majority being severe depressive, went through an intervention of exercise and healthy diet. The results, measured every 3 months up to 9 months, showed, compared to baseline, increased physical capacity, decreased waist measurements, and a significant decrease in mental symptoms.

As a conclusion, earlier research has explored the effects of health-related habits such as physical activity, diet and social support on depression and anxiety, but rarely in a comorbid approach and not as a comprehensive treatment including all elements. More research on groups with comorbid depression and anxiety is needed as it is a relatively unexplored area (Bandelow et al., 2017).

Following from this, the aim of the present study was to explore the longitudinal effects of a comprehensive treatment, including physical exercise, nutrition, social training, and psychoeducation, in addition to traditional psychopharmacology and psychotherapy, on patients with comorbid anxiety and depression in a clinical context.

## 2 Material and method

This is a longitudinal, intervention study (Caruana et al., 2015) where anxiety and depressive symptoms were measured and formed a basis for classification of comorbid symptomatology and variations across time. There were three measure occasions, at baseline, in the end of treatment and 3 months after ended treatment. The method is further described in the following sections.

### 2.1 The clinical context

The participants were inpatients at a foundation-driven psychiatric clinic in Norway. The treatment lasted for 12 weeks in most cases. Requirements for staying at the clinic were being adult (age > 18), no serious symptoms of psychosis, no suicidal behavior, no substance abuse, and no self-harm or violence against others. Approximately 95 per cent of the patients had experiences of trauma and often a long history in psychiatric care. They were referred to the clinic by specialist psychiatry. In most cases, treatment targeted anxiety and depression, e.g., psychotherapy, individually and in groups, as well as pharmacological treatment. The psychotherapy methods used in individual therapy were mainly Intensive Short-Term Dynamic Psychotherapy (ISTDP) (Ecker et al., 2024) and Eye Movement Desensitization Reprocessing Therapy (EMDR) (de Jongh et al., 2024). The therapy was performed by a psychologist. In addition to this, psychologists and specialist nurses practiced individual psychodynamic, cognitive, and affect-focused forms of therapy.

The group therapy was dominated by psychodynamic (Messer and McWilliams, 2007), psychoeducational (Donker et al., 2009), and eclectic (Vasile, 2021) forms of therapy and were given by two psychiatric nurse group therapists.

In addition to this, patients were encouraged to participate in a minimum of three sessions a week of physical activity offered at the clinic. Examples of physical activity were walking, resistance training, horseback riding, climbing, and boxing. Duration of activities were varying and, if possible, customized to the patient, i.e., patients unusual to physical training may have had difficulties baseline to perform intensive training and a walk was enough. During their stay, the patients were exhorted to participate in social contexts at the clinic, as meal preparation, where they also gained knowledge in preparing healthy food. Through social interactions at the clinic, such as shared meals and group activities social training was performed. The patients' social context was emphasized and their social relationships and relatives were regarded as important resources in the treatment. If needed patients could have individual support concerning social training.

An intensive care nurse with a master's degree in public health was responsible for the different kinds of physical activities, trauma sensitive yoga, and food preparations.

The patients were offered different types of psychoeducation (Donker et al., 2009), for example learning more about different reactions to anxiety and what causes them, what healthy lifestyle habits can look like and what impact they have on mental health.

In summary, the treatment was person-centered and with a focus on the patients' resources, rather than his or her weaknesses, to achieve better mental health and improved life quality.

### 2.2 Participants and procedure

The current investigation is a longitudinal intervention study. Respondents were inpatients on the psychiatric clinic and were asked to participate in the study when they arrived at the clinic during the period January 2019 to January 2023. Beside the requirements to stay at the clinic no further exclusion criteria were used. Respondents were fully informed about the aim of the study and 176 individuals gave their consent to participate. No patients declining participation were reported. Each patient received a tablet computer when they got to the clinic and by using a personal code the respondents got access to the questionnaires. Data collection was carried out a total of three times; two times during their stay at the clinic, week 1 and 11, and one time 3 months after leaving the clinic.

The group (*n* = 176) consisted of adult (>18 years) men (*n* = 39) and women (*n* = 137) with a mean age of 40.1 years (*SD* = 12.2) with mild to severe anxiety and/or depression. Among these, 80 respondents (men *n* = 15, women *n* = 65), mean age 43.8 (SD = 11.55) participated in all three data collections. Descriptive analysis of the respondent's demographic background showed that a majority of the respondents were women (77.8%). Half of the respondents were married or living together with someone (50.3%). Approximately two thirds (58.6%) had primary-/senior high school as highest education and one third had education from university. A majority were not employed (79.8%).

### 2.3 Measures

Beck Anxiety Inventory (BAI) measures anxiety in clinical contexts. The participant self-estimates the occurrence of anxiety symptoms during the last week in 21 symptom descriptions, for example “unable to relax” and “fear of worst.” The scale runs from 0 = *not at all*, to 3 = *a lot*. The instrument is well established and translated into many languages (Beck et al., 1988a). The Cronbach alpha in the present study was T1 = 0.92, T2 = 0.84, T3 = 0.92. The single answers from each person were summarized into an index on each measurement occasions. The index was then divided into three categories [0–21 = 1, mild; 22–35 = 2, moderate; and 36–63 = 3, severe (Saal et al., 2019)].

Beck Depression Inventory II (BDI-II) measures depression in clinical contexts. The instrument consists of 21 statements of depression on varying degrees, e.g., “sad.” The patient assesses the occurrence during the last 2 weeks, according to a scale of 0–3, where 0 = “*I do not feel sad”* and 3 = “*I am so sad and unhappy that I can't stand it”* (Beck et al., 1988b). The Cronbach alpha in the present study was T1 = 0.93, T2 = 0.90. T3 = 0.93. An index was created summarizing the single answers from each person on each measurement occasions, and then categorized based on the BDI total score [0–13 = 1, minimal; 14–19 = 2, mild; 20–28 = 3, moderate; and 29–63 = 4, severe (Beck et al., 1988b)].

Before the analysis, the BAI and BDI categories were dichotomized were the BAI category 1 (mild) was scored as 0 and the BAI categories 2–3 (moderate and severe) were scored as 1. The BDI went through the same process, were the BDI categories 1–2 (minimal and mild) were scored as 0 and 3–4 (moderate and severe) were scored as 1.

The dichotomized variables were then cross-tabulated to compose four comorbid depression and anxiety groups [(BAI_dic = 0 + BDI_dic = 0) = 0]; [(BAI_dic = 1 + BDI_dic =0) = 1]; [(BAI_dic = 0 + BDI_dic = 1) =2]; and [(BAI_dic + BDI_dic = 1) = 3]. This means that an individual with mild anxiety and minimal/mild depression was placed in group 0 and a person with severe anxiety and severe depression was placed in group 3.

### 2.4 Data analysis

The Statistical Package for the Social Sciences (SPSS) 27.0 was used to perform the analysis and *p*-values < 0.05 were considered as statistically significant. Shapiro-Wilk test and plots were used to judge if the data were normally distributed, and these indicated not normal distributions and hence non-parametric tests were used. Mann Whitney and chi-square tests were used to analyze drop out and demographic variables. The Friedmans test and the McNemar test were used to analyze the longitudinal data.

### 2.5 Ethics

The project has been reviewed and approved by the Norwegian Regional Committees for Medical and Health Research Ethics (REK) (#20009). The participants were informed about the aim of the project and that the participation in the study was voluntary and that they could withdraw their written consent at any time without telling why. They were informed that withdrawal would not affect their treatment.

## 3 Results

### 3.1 Dropout analysis

To investigate whether there were differences between those who participated in all three data collections points and those who did not, non-response bias analyses were performed. These did not show any differences regarding demographic variables. Responses to the BAI and the BDI from the first measurement were compared between those who answered all three measurements and those who dropped out. There were no significant differences between the groups {Mann-Whitney, BAI, dropout [*Median (Md)* =*22.0, n* = 69] non-dropouts (*Md* = 24, *n* = 86), *z* = 1.372, *p* = 0.17. BDI, dropout (*Md* = 28.0, *n* = 69) non-dropout (*Md* = 28.0, *n* = 80), z = 1.358, *p* = 0.18}.

### 3.2 Treatments effect on anxiety and depression

Table 1 presents the BAI and BDI summarized indices of the respondent's answers in the beginning of the treatment (week 1), at the end of the treatment (week 11) and 3 months after (week 24). There were no statistically significant changes.

**Table 1:** Summarized index BAI and BDI, mean (SD) and median (interval^a^).

**Measure week**	**BAI mean (SD)**	**BAI median (interval)**	**BDI mean (SD)**	**BDI median (interval)**
1	26.48 (13.39)	24.5 (15.25–36.0)	29.42 (12.13)	28.0 (22.0–39.0)
11	25.75 (12.95)	23.0 (16.0–33.75)	28.18 (13.10)	29.5 (18.25–40.0)
24	24.25 (12.83)	21.5 (16.0–31.75)	28.04 (13.66)	28.0 (17.0–39.0)

Friedmans test; BAI, chi-squared (2*, n* = 80) = 3.282, *p* = 0.30, BDI chi-squared (2*, n* = 80) = 0.438, *p* = 0.80. ^a^25 percentile−75 percentile.

### 3.3 Treatment effects on comorbidity

**Figure 1:**
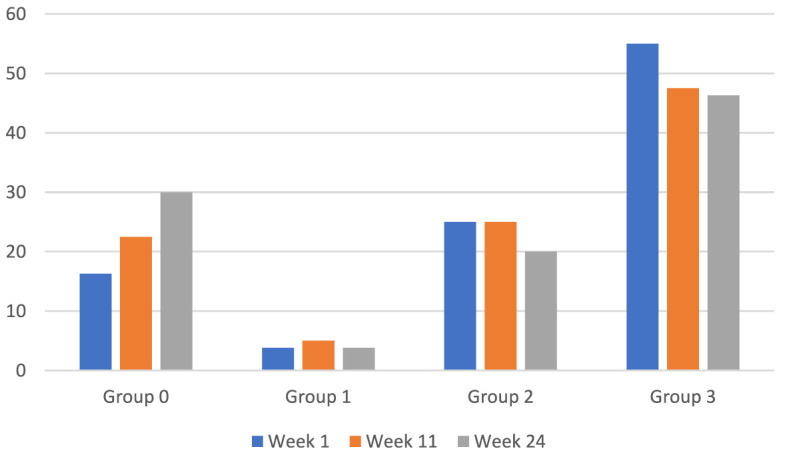
Longitudinal changes in frequencies within BAI/BDI based groups. Based on Beck Anxiety Inventory (BAI) and Beck Depression Inventory (BDI). Group 0 = low anxiety and low depression, group 1 = high anxiety and low depression, group 2 = low anxiety and high depression, and group 3 = high anxiety and high depression. Total *n* = 80.

To investigate the effects of the treatment on comorbid anxiety and depression the instruments BAI and BDI were combined to create four groups: group 0 contained those with mild anxiety and mild depression, group 1 severe anxiety and mild depression, group 2 mild anxiety and severe depression, and group 3 severe anxiety and severe depression (see Figure 1).

The number of patients in group 0 (mild anxiety and mild depression) increased by 46% (13 vs. 24 individuals) in week 24 compared to week 1, which was a significant change (McNemar, χ^2^(1*, n* = 80) = 4.348, *p* = 0.035).

### 3.4 Additional comparisons based on demographic characteristics

The analysis revealed significant differences in BAI and BDI scores in relation to demographic variables. When measured at T1, women had a higher incidence of anxiety compared to men [Mann Whitney males (*Md* = 19.0, *n* = 17) female (*Md* = 26.0, *n* = 69), *z* = 2.2, *p* = 0.027]. In terms of education level, there were significant differences in BAI baseline; those who had primary/secondary school as their highest education, had a higher incidence of anxiety compared to those who had university education [Mann Whitney, primary/secondary school (*Md* = 31.5, *n* = 50) university (*Md* = 26.6, *n* = 30), *z* = −2.192 *p* = 0.028]. No statistically significant differences were observed baseline in age, marital status or employment.

### 3.5 Summary of results

To summarize the results, it can be concluded that through the comprehensive treatment there was an increase in the group with mild depression and mild anxiety across the measurement occasions. Comparisons based on demographic variables showed a higher prevalence of anxiety among women than men and a higher prevalence of anxiety among those with primary/secondary school as their highest education compared to those with university education.

## 4 Discussion

In this section we will first address the aim of the study and how the results respond to it, we further discuss implications of the result on treatment and the mental health of this patients. Following, are methodological considerations where we discuss weaknesses and strength of the study. The section end with practical implications and conclusions.

The aim of the present study was to explore the longitudinal effects of a comprehensive treatment, including physical exercise, nutrition, social training, and psychoeducation, in addition to traditional psychopharmacology and psychotherapy, on patients with comorbid anxiety and depression in a clinical context. The result of the longitudinal analysis showed that the group with low anxiety and low depression increased significantly during the study. This means that individuals who in previous measurements had a higher incidence of anxiety and/or depression, at the end of the study had reduced levels 12 weeks after leaving the clinic. No other statistically significant differences were found, although a non-significant reduction of group 3 (severe anxiety and severe depression) can be noted.

Finding significant decreases in comorbid anxiety and depression is promising in a context of comorbidity as a difficult group to treat. Studying comorbid cases of anxiety and depression is important as the diagnoses often occur together in varying degrees (Lamers et al., 2011).

Analysis of demographics is in line with previous research (WHO, 2023a). Female respondents had a higher prevalence of anxiety and respondents with primary/secondary school as highest education reported higher prevalence of anxiety and depression. According to Macintyre et al. (2018) lower socioeconomic groups have a higher prevalence of poorer mental health. A reason to this may be a current stress concerning employment, housing and lower income (Hudson, 2005).

Many of the participants had extensive previous experience with various types of psychiatric institutions and treatments. The majority had traumatic experiences, which might make symptoms and treatment even more complex. Earlier research has explored the effects of physical activity (Andersson et al., 2022; Merom et al., 2008; Singh et al., 2023), healthy nutrition (Owen and Corfe, 2017), social support and psychoeducation (Lean et al., 2019), but often including only one of the elements. In current study we explore the effects of a treatment intervention were all these elements are included. Given the comprehensive treatment, it is difficult to know which part of the treatment that brought the improved mental wellbeing in the group. Perhaps one of the parts is more contributing than one others, but we believe that it is the comprehension itself that cause the effects. There may also be bidirectional effects between the three parts that create a synergy resulting in a total effect on mental health that is greater than the sum of the parts on their own. Perhaps it can also be, as Rolin et al. (2020) states, that a treatment that takes several dimensions of a person into account not only leads to better physical and mental health but can also lead to a feeling of being able to influence one's own mood and situation. The person-centered treatment including a healthy lifestyle might give the patient a sense of control, manageability, and meaningfulness, aspects that can have a major impact on mental wellbeing (Antonovsky, 1987). A conclusion of that may be that the treatment can have good long-term effects on the individuals' self-image and the situations they face later in life.

### 4.1 Methodological considerations

A possible weakness in the study is that we have no information of the patients' contextual situation when they leave the clinic and in what way this affect the mental health. Coming home to an everyday life with unchanged structure may lead to a regression to old habits and not being able to uphold the new ones.

Although there were longitudinal decreases in BDI and BAI scores at nominal level, there were no statistically significant effects, possible due to a lack of statistical power. There was a relatively large dropout between the first and last data collection time point resulting in a moderate sample size. Unfortunately, this is a common problem in longitudinal studies (Caruana et al., 2015). However, dropout analyzes showed no significant differences among those who responded to baseline and then dropped out during the study compared to those who responded to all three measurements. It shows that those who completed the study did not have more or less symptoms of depression or anxiety compared to those who did not. The longitudinal design of this study is a strength and allows for investigation of anxiety and depressive symptoms throughout the treatment.

This study also contains some other potential weaknesses that must be considered when interpreting the results as these might limit the generalizability and cause bias of the findings. First, the skewed gender ratio, women and men may respond different to various treatment alternatives. Though, seen in a historical way the situation has been the opposite, research concerning men have dominated, and Mizock and Brubaker (2021) urge the importance of further exploring women experience of care and treatment concerning mental illness.

Second, the absence of a control group. An experimental design with a randomized control study is considered being the gold standard when it comes to evaluating effects of an intervention (Zabor et al., 2020). The use of such a research design in present study was unfortunately not possible as the study was carried out in a psychiatric clinic offering this type of treatment to all patients. A possible solution to this may have been to include a group of patients only perceiving psychotherapy and pharmacology, but this treatment would have to been performed at another clinic, which in turn entails that other variations in the treatment or in the context could impact the results.

Third, both instruments are self-reported questionnaires, which may lead to over- or underestimation of measured phenomena (Lira et al., 2022). The instruments are though well established, and the measures of internal consistency in the current study were high (all measure occasions over 0.80). Together with the longitudinal design this brings good validity and reliability.

### 4.2 Practical implications

We recommend that clinics working with comorbid depression and anxiety patients should add physical activity, nutrition advice, and psychoeducation to the traditional treatment regimes. However, to implement such a multifaceted program in a clinical setting will require additional costs and efforts to secure the right expertise and organize the necessary practical aspects. But as mental illness is rapidly increasing worldwide it is of the outmost importance to implement alternative treatment methods to slow down, or even decrease, the prevalence of mental illness.

This area is sparsely researched, and more focus is needed to help this vulnerable group gain a better mental health and quality of life.

## 5 Conclusion

The main conclusion of this study is that through the comprehensive (pharmacological, psychological, physical, and social), person-centered treatment the group with mild anxiety and depression significantly increased through the treatment. More knowledge about clinical applications of non-pharmacological treatment alternatives for patients with comorbid depression and anxiety will expand the treatment possibilities. Earlier studies have shown beneficial effects of physical activity, optimal nutrition, and other interventions, but for the most part individually, on each mental illness. The current study shows positive results for a multifaceted approach encompassing several lifestyle aspects that improve mental illness during treatment, and that are also important habits that may carried forward in life.

Future studies are recommended to further explore alternative treatments concerning comorbid anxiety and depression, and, especially, the long-term effects on mental wellbeing and quality of life.

## Data Availability

The raw data supporting the conclusions of this article will be made available by the authors, without undue reservation.

## References

[B1] American Psychiatric Association (2013). Diagnostic and statistical manual of mental disorders: DSM-5™, 5th ed. Arlington, VA, US: American Psychiatric Publishing, Inc. 10.1176/appi.books.9780890425596

[B2] Andersson E. Hovland A. Kjellman B. Taube J. Martinsen E. (2015). Fysisk aktivitet lika bra som KBT eller läkemedel vid depression. Läkartidningen 47, 2102–2104. 26574804

[B3] Andersson E. Hovland A. Taube J. Kjellman B. Hedlund L. Martinsen E. W. (2022). Fysisk aktivitet kan ha omedelbar effekt på depression och ångest. Läkartidningen 3. 36106742

[B4] Antonovsky A. (1987). Unraveling the mystery of health: how people manage stress and stay well. San Francisco 175.

[B5] Bandelow B. Michaelis S. Wedekind D. (2017). Treatment of anxiety disorders. Dial. Clin. Neurosci. 19, 93–107. 10.31887/DCNS.2017.19.2/bbandelow 28867934 PMC5573566

[B6] Beauregard N. Marchand A. Blanc M.-E. (2011). What do we know about the non-work determinants of workers' mental health? A systematic review of longitudinal studies. BMC Public Health 11, 1–15. 10.1186/1471-2458-11-439 21645393 PMC3141446

[B7] Beck A. T. Epstein N. Brown G. Steer R. A. (1988a). An inventory for measuring clinical anxiety: psychometric properties. J. Consult. Clin. Psychol. 56:893. 10.1037//0022-006X.56.6.893 3204199

[B8] Beck A. T. Steer R. A. Carbin M. G. (1988b). Psychometric properties of the beck depression inventory: twenty-five years of evaluation. Clin. Psychol. Rev. 8, 77–100. 10.1016/0272-7358(88)90050-5

[B9] Bond G. Stanton R. Wintour S.-A. Rosenbaum S. Rebar A. L. (2020). Do exercise trials for adults with depression account for comorbid anxiety? A systematic review. Ment. Health Phys. Act. 18:100320. 10.1016/j.mhpa.2020.100320

[B10] Buckman J. E. Saunders R. O'Driscoll C. Cohen Z. D. Stott J. Ambler G.. (2021). Is social support pre-treatment associated with prognosis for adults with depression in primary care? Acta Psychiatr. Scand. 143, 392–405. 10.1111/acps.13285 33548056 PMC7610633

[B11] Caruana E. J. Roman M. Hernández-Sánchez J. Solli P. (2015). Longitudinal studies. J. Thorac. Dis. 7:E537. 10.3978/j.issn.2072-1439.2015.10.63 26716051 PMC4669300

[B12] Chen C. (2022). Recent advances in the study of the comorbidity of depressive and anxiety disorders. Adv. Clin. Exper. Med. 31, 355–358. 10.17219/acem/147441 35394125 PMC13539099

[B13] Cooney G. M. Dwan K. Greig C. A. Lawlor D. A. Rimer J. Waugh F. R.. (2013). Exercise for depression. Cochr. Datab. System. Rev. 2:4366. 10.1002/14651858.CD004366.pub6 24026850 PMC9721454

[B14] de Jongh A. de Roos C. El-Leithy S. (2024). State of the science: Eye movement desensitization and reprocessing (EMDR) therapy. J. Traum. Stress. 37, 205–216. 10.1002/jts.23012 38282286

[B15] de Oliveira Penaforte F. R. Minelli M. C. S. Anastácio L. R. Japur C. C. (2019). Anxiety symptoms and emotional eating are independently associated with sweet craving in young adults. Psychiatry Res. 271, 715–720. 10.1016/j.psychres.2018.11.070 30791346

[B16] Donker T. Griffiths K. M. Cuijpers P. Christensen H. (2009). Psychoeducation for depression, anxiety and psychological distress: a meta-analysis. BMC Med. 7, 1–9. 10.1186/1741-7015-7-79 20015347 PMC2805686

[B17] Dunner D. L. (2001). Management of anxiety disorders: the added challenge of comorbidity. Depress. Anxiety 13, 57–71. 10.1002/da.1018 11301922

[B18] Ecker B. Ticic R. Hulley L. (2024). “Intensive short-term dynamic psychotherapy (ISTDP),” in Unlocking the Emotional Brain (London: Routledge). 10.4324/9781003231431

[B19] Gibson-Smith D. Bot M. Brouwer I. A. Visser M. Penninx B. W. (2018). Diet quality in persons with and without depressive and anxiety disorders. J. Psychiatr. Res. 106, 1–7. 10.1016/j.jpsychires.2018.09.006 30240962

[B20] Goodwin G. M. (2021). Revisiting treatment options for depressed patients with generalised anxiety disorder. Adv. Ther. 38, 61–68. 10.1007/s12325-021-01861-0 34417993 PMC8437852

[B21] Grey I. Arora T. Thomas J. Saneh A. Tohme P. Abi-Habib R. (2020). The role of perceived social support on depression and sleep during the COVID-19 pandemic. Psychiatry Res. 293:113452. 10.1016/j.psychres.2020.113452 32977047 PMC7500407

[B22] Hanna K. Cross J. Nicholls A. Gallegos D. (2023). The association between loneliness or social isolation and food and eating behaviours: a scoping review. Appetite 191:107051. 10.1016/j.appet.2023.107051 37802217

[B23] Hearing C. Chang W. Szuhany K. Deckersbach T. Nierenberg A. Sylvia L. G. (2016). Physical exercise for treatment of mood disorders: a critical review. Curr. Behav. Neurosci. Rep. 3, 350–359. 10.1007/s40473-016-0089-y 28503402 PMC5423723

[B24] Hudson C. G. (2005). Socioeconomic status and mental illness: tests of the social causation and selection hypotheses. Am. J. Orthopsych. 75, 3–18. 10.1037/0002-9432.75.1.3 15709846

[B25] Ironside M. DeVille D. C. Kuplicki R. T. Burrows K. P. Smith R. Teed A. R.. (2023). The unique face of comorbid anxiety and depression: increased interoceptive fearfulness and reactivity. Front. Behav. Neurosci. 16:1083357. 10.3389/fnbeh.2022.1083357 36755667 PMC9899910

[B26] Lai J. S. Hiles S. Bisquera A. Hure A. J. McEvoy M. Attia J. (2014). A systematic review and meta-analysis of dietary patterns and depression in community-dwelling adults. Am. J. Clin. Nutr. 99, 181–197. 10.3945/ajcn.113.069880 24196402

[B27] Lamers F. van Oppen P. Comijs H. C. Smit J. H. Spinhoven P. van Balkom A. J.. (2011). Comorbidity patterns of anxiety and depressive disorders in a large cohort study: the netherlands study of depression and anxiety (NESDA). J. Clin. Psychiat. 72:341. 10.4088/JCP.10m06176blu 21294994

[B28] Lean M. Fornells-Ambrojo M. Milton A. Lloyd-Evans B. Harrison-Stewart B. Yesufu-Udechuku A.. (2019). Self-management interventions for people with severe mental illness: systematic review and meta-analysis. Br. J. Psychiat. 214, 260–268. 10.1192/bjp.2019.54 30898177 PMC6499726

[B29] Leonard J. (2020). Anxiety and Loss of Apetite-What is the Link? Medical News Today.

[B30] Li Y. Lv M.-R. Wei Y.-J. Sun L. Zhang J.-X. Zhang H.-G.. (2017). Dietary patterns and depression risk: a meta-analysis. Psychiatry Res. 253, 373–382. 10.1016/j.psychres.2017.04.020 28431261

[B31] Lira B. O'Brien J. M. Peña P. A. Galla B. M. D'Mello S. Yeager D. S.. (2022). Large studies reveal how reference bias limits policy applications of self-report measures. Sci. Rep. 12:19189. 10.1038/s41598-022-23373-9 36357481 PMC9649615

[B32] Macintyre A. Ferris D. Gonçalves B. Quinn N. (2018). What has economics got to do with it? The impact of socioeconomic factors on mental health and the case for collective action. Palgr. Communic. 4, 1–5. 10.1057/s41599-018-0063-2

[B33] Merom D. Phongsavan P. Wagner R. Chey T. Marnane C. Steel Z.. (2008). Promoting walking as an adjunct intervention to group cognitive behavioral therapy for anxiety disorders—a pilot group randomized trial. J. Anxiety Disord. 22, 959–968. 10.1016/j.janxdis.2007.09.010 17988832

[B34] Messer S. B. McWilliams N. (2007). “Insight in psychodynamic therapy: theory and assessment,” in Insight in psychotherapy, ed. L. G. Castonguay and C. Hill (London: American Psychological Association), 9–29. 10.1037/11532-001

[B35] Mizock L. Brubaker M. (2021). Treatment experiences with gender and discrimination among women with serious mental illness. Psychol. Serv. 18:64. 10.1093/med-psych/9780190922351.001.0001 30907615

[B36] Owen L. Corfe B. (2017). The role of diet and nutrition on mental health and wellbeing. Proc. Nutr. Soc. 76, 425–426. 10.1017/S0029665117001057 28707609

[B37] Paans N. P. Bot M. van Strien T. Brouwer I. A. Visser M. Penninx B. W. (2018). Eating styles in major depressive disorder: results from a large-scale study. J. Psychiatr. Res. 97, 38–46. 10.1016/j.jpsychires.2017.11.003 29175296

[B38] Rebar A. L. Stanton R. Rosenbaum S. (2017). Comorbidity of depression and anxiety in exercise research. Lancet Psychiat. 4:519. 10.1016/S2215-0366(17)30164-5 28652042

[B39] Roer G. E. Solbakken H. H. Abebe D. S. Aaseth J. O. Bolstad I. Lien L. (2021). Inpatients experiences about the impact of traumatic stress on eating behaviors: an exploratory focus group study. J. Eating Disor. 9, 1–12. 10.1186/s40337-021-00480-y 34565487 PMC8474934

[B40] Rolin D. Fox I. Jain R. Cole S. P. Tran C. Jain S. (2020). Wellness interventions in psychiatrically ill patients: impact of WILD 5 wellness, a five-domain mental health wellness intervention on depression, anxiety, and wellness. J. Am. Psychiatr. Nurses Assoc. 26, 493–502. 10.1177/1078390319886883 31738111

[B41] Saal W. L. Kagee A. Bantjes J. (2019). Evaluation of the beck anxiety inventory in predicting generalised anxiety disorder among individuals seeking HIV testing in the Western Cape province, South Africa. South African J. Psychiat. 25, 1–5. 10.4102/sajpsychiatry.v25i0.1336 31824741 PMC6890543

[B42] Shao R. He P. Ling B. Tan L. Xu L. Hou Y.. (2020). Prevalence of depression and anxiety and correlations between depression, anxiety, family functioning, social support and coping styles among Chinese medical students. BMC Psychol. 8, 1–19. 10.1186/s40359-020-00402-8 32321593 PMC7178943

[B43] Simmons W. K. Burrows K. Avery J. A. Kerr K. L. Bodurka J. Savage C. R.. (2016). Depression-related increases and decreases in appetite: dissociable patterns of aberrant activity in reward and interoceptive neurocircuitry. Am. J. Psychiat. 173, 418–428. 10.1176/appi.ajp.2015.15020162 26806872 PMC4818200

[B44] Singh B. Olds T. Curtis R. Dumuid D. Virgara R. Watson A.. (2023). Effectiveness of physical activity interventions for improving depression, anxiety and distress: an overview of systematic reviews. Br. J. Sports Med. 57, 1203–1209. 10.1136/bjsports-2022-106195 36796860 PMC10579187

[B45] Terry R. Townley G. (2019). Exploring the role of social support in promoting community integration: an integrated literature review. Am. J. Community Psychol. 64, 509–527. 10.1002/ajcp.12336 31116874

[B46] Theal R. Tay V. X. P. Hickman I. J. (2018). Conflicting relationship between dietary intake and metabolic health in PTSD: a systematic review. Nutr. Res. 54, 12–22. 10.1016/j.nutres.2018.03.002 29914663

[B47] Van Citters A. D. Pratt S. I. Jue K. Williams G. Miller P. T. Xie H.. (2010). A pilot evaluation of the In SHAPE individualized health promotion intervention for adults with mental illness. Commun. Ment. Health J. 46, 540–552. 10.1007/s10597-009-9272-x 20012197 PMC3163497

[B48] Vasile C. (2021). Eclectic psychotherapy and case formulation. J. Educ. Sci. Psychol. 11, 162–168. 10.51865/JESP.2021.2.18

[B49] WHO (2023a). Anxiety Disorders. Available at: https://www.who.int/news-room/fact-sheets/detail/anxiety-disorders (accessed November 23, 2020).

[B50] WHO (2023b). Depressive Disorder. Available at: https://www.who.int/news-room/fact-sheets/detail/depression (accessed November 23, 2020).

[B51] Zabor E. C. Kaizer A. M. Hobbs B. P. (2020). Randomized controlled trials. Chest 158, S79–S87. 10.1016/j.chest.2020.03.013 32658656 PMC8176647

[B52] Zhang X. Ravichandran S. Gee G. C. Dong T. S. Beltrán-Sánchez H. Wang M. C.. (2024). Social isolation, brain food cue processing, eating behaviors, and mental health symptoms. JAMA Netw. Open 7:e244855. 10.1001/jamanetworkopen.2024.4855 38573637 PMC11192185

[B53] Zhiguo W. Yiru F. (2014). Comorbidity of depressive and anxiety disorders: challenges in diagnosis and assessment. Shanghai Arch. Psychiat. 26:227. 10.3969/j.issn.1002-0829.2014.04.006 25317009 PMC4194005

